# Transcriptome atlases of rat brain regions and their adaptation to diabetes resolution following gastrectomy in the Goto-Kakizaki rat

**DOI:** 10.1186/s13041-025-01176-z

**Published:** 2025-02-07

**Authors:** François Brial, Aurélie Le Lay, Claude Rouch, Edouard Henrion, Mathieu Bourgey, Guillaume Bourque, Mark Lathrop, Christophe Magnan, Dominique Gauguier

**Affiliations:** 1 Université Paris Cité, INSERM U1132 Biologie de l’os et du Cartilage (BIOSCAR), 75010 Paris, France; 2 Université Paris Cité, Functional and Adaptive Biology, UMR 8251, CNRS, 4 Rue Marie Andrée Lagroua Weill-Halle, 75013 Paris, France; 3 Victor Phillip Dahdaleh Institute of Genomic Medicine at McGill University, 740 Doctor Penfield Avenue, Montreal, QC H3A 0G1 Canada; 4 Center for Genomic Medicine, Kyoto University Graduate School of Medicine, Kyoto, 606-8501 Japan

**Keywords:** Bariatric surgery, Vertical sleeve gastrectomy, RNA sequencing

## Abstract

Brain regions drive multiple physiological functions through specific gene expression patterns that adapt to environmental influences, drug treatments and disease conditions. To generate a detailed atlas of the brain transcriptome in the context of diabetes, we carried out RNA sequencing in hypothalamus, hippocampus, brainstem and striatum of the Goto-Kakizaki (GK) rat model of spontaneous type 2 diabetes, which was applied to identify gene transcription adaptation to improved glycemic control following vertical sleeve gastrectomy (VSG) in the GK. Over 19,000 distinct transcripts were detected in the rat brain, including 2794 which were consistently expressed in the four brain regions. Region-specific gene expression was identified in hypothalamus (n = 477), hippocampus (n = 468), brainstem (n = 1173) and striatum (n = 791), resulting in differential regulation of biological processes between regions. Differentially expressed genes between VSG and sham operated rats were only found in the hypothalamus and were predominantly involved in the regulation of endothelium and extracellular matrix. These results provide a detailed atlas of regional gene expression in the diabetic rat brain and suggest that the long term effects of gastrectomy-promoted diabetes remission involve functional changes in the hypothalamus endothelium.

**Supplementary Information:**

The online version contains supplementary material available at 10.1186/s13041-025-01176-z.

## Introduction

The mammalian brain is characterised by a networked organisation of regions which orchestrate multiple biological functions [1]. Functional connections between the central nervous system (CNS) and pancreatic islets [2, 3] and the gastrointestinal tract [4, 5] regulate glucose homeostasis and body weight and, when disrupted, result in type 2 diabetes (T2D) and obesity. Recent research has shown that bariatric surgery, which is primarily used in the treatment of obesity [6, 7], results in improved glucose homeostasis and even T2D remission through mechanisms that are at least in part independent of weight loss and reduced caloric intake [8]. Gastrectomy-promoted improvement in metabolic profile occurs in the absence of weight loss in mice and rats [9, 10] and often precedes weight loss [11]. Understanding the underlying biological mechanisms will provide important information in novel treatment algorithms of T2D care [12].

A contribution of glucagon-like peptide 1 (GLP-1) [13], the gut microbiota [14] and the metabolism of bile acids [15] to gastrectomy-promoted T2D remission has been proposed.

The most commonly used bariatric surgery techniques involve invasive methods [16] that disrupt essential hormonal and neural signals between the CNS and the gastrointestinal tract [17]. The ventral and dorsal gastric branches of the vagus nerve that reach the pancreas are severed by gastrectomy [17]. Vagotomy and sympathectomy may also result in reduced gastric secretion of ghrelin [18] and neuronal production of GLP-1 from the brainstem [19]. Bariatric surgery affects the regulation of multiple gut peptide hormones, including mainly ghrelin, cholecystokinin, the peptide tyrosine–tyrosine, GLP-1, the gastric inhibitory polypeptide and neurotensin, and subsequently alters gut-brain signalling mechanisms [20]. These complex regulatory processes underline the important metabolic consequences of sectioning gastric innervation and altering neuroendocrine mechanisms in gastrectomised individuals.

Preclinical models of T2D provide powerful experimental systems to analyse the physiological consequences of bariatric surgery and to advance our understanding of the molecular mechanisms contributing to improved glucose homeostasis. We and others have demonstrated that gastrectomy results in improved glucose homeostasis in Otsuka Long-Evans Tokushima Fatty (OLETF) rats [21], in the Goto-Kakizaki (GK) rat model of lean T2D [22–28], in rats injected with streptozotocin [23] and in mouse models of T2D and obesity caused by high fat diet feeding [29]. We showed that permanent reduction in glucose intolerance in the GK following vertical sleeve gastrectomy (VSG) is associated with changes in the metabolism of bile acids and in the architecture of the gut microbiota dominated by intestinal enrichment of *Prevotella copri* [22]. To test the hypothesis of a remodelling of brain gene expression in response to gastrectomy-promoted improvement of glucose homeostasis, we generated a detailed transcriptome atlas of the diabetic brain using hypothalamus, hippocampus, brainstem and striatum samples from the GK rat. We applied this resource to the identification of genes differentially expressed between VSG-treated and sham operated GK rats, which pointed to altered regulation of the hypothalamus endothelium and extracellular matrix.

## Material and methods

### Animals

Inbred Goto-Kakizaki (GK/Ox colony) rats were bred in our animal facility in individually ventilated cages. Rats were maintained in a controlled environment with 12 h dark–light cycles, a temperature of 22–24 °C, and a relative humidity of 50–60%. Rats had access to water and standard rat chow (SAFE, Augy, France) ad libitum. Animal procedures were authorized by a licence (Ref. 4231 201602231507187) under the Charles Darwin Ethics Committee in Animal Experiment, Paris, France.

### Vertical sleeve gastrectomy (VSG)

12-week-old male GK rats were anesthetized by isoflurane intoxication. The lateral 80% of the stomach was excised with a linear cutter (TLC55, Ethicon, Issy Les Moulineaux, France) to leave a tubular gastric remnant in continuity with the oesophagus, the pylorus and the duodenum. Enrofloxacine 2.5% (5 mg/kg body weight) and buprenorphine (200 µg/kg body weight) were applied for three days for post-surgical analgesia. A sham operation was carried out in GK controls involving application of pressure with blunt forceps along a vertical line between the oesophageal sphincter and the pylorus. Body weight and blood glucose were regularly recorded in both gastrectomised and sham operated rats over a period of 136 days after surgery.

### Sample collection

Overnight fasted gastrectomised and sham operated rats were killed 140 days after surgery by lethal injection of sodium pentobarbital. Four brain regions (hypothalamus, hippocampus, brainstem, striatum) were carefully dissected, snap-frozen in liquid nitrogen and stored at − 80 °C.

### RNA preparation and sequencing pipeline

RNA from the four brain regions of gastrectomised and sham operated GK rats was prepared using the RNeasy RNA Mini Kit (Qiagen, Courtaboeuf, France), fragmented and converted to cDNA. The cDNA was end-repaired, A-tailed and adapter-ligated before amplification and size selection. Sequencing libraries were prepared, multiplexed and quality controlled prior to 51-nt paired end sequencing on an Illumina HiSeq2000.

The GenPipe suite of programmes was used for processing RNA-sequencing raw reads [30]. Briefly, reads were trimmed from the 3’ end to have a Phred score of over 30 and filtered for a minimum length of 32 bp. Clipping Illumina sequencing adapters was performed using Trimmomatic v. 0.36 [31]. Filtered reads were aligned to the rat genome reference Rnor_6.0using STAR v. 2.5.3 with 2-passes mode [32] which created Binary Alignment Map files (.bam). Raw read counts of Ensembl genes (version 84) were obtained using HTseq-count v. 0.6.1 [33]. Assembly of aligned RNA-Seq reads into transcripts and abundance estimates in Fragments Per Kilobase of exon per Million fragments mapped (FPKM) were generated with the Cufflinks program [34]. Consistency of sequencing data between biological replicates was verified for each brain region by pairwise Pearson's correlation analysis. Exploratory analysis techniques were applied to data quantified by the HTseq calculated counts-per-million reads (log2CPM) and cufflinks estimates (log2FPKM) in order to detect possible outliers or mislabelling and to explore the homogeneity of biological replicates. Connection between samples was evaluated by hierarchical clustering on the log2(CPM) and by both principal component analysis (PCA) and multi-dimensional scaling (MDS) plots.

Raw RNA sequencing data are available through the GenBank Sequence Read Archive (SRA) under the project reference ERP166514: https://trace.ncbi.nlm.nih.gov/Traces/?view=study&acc=ERP166514

### Statistical analysis of differential expression

Differential expression analyses at gene level were performed on normalized read counts using DESeq2 [35] and edgeR [36] R Bioconductor packages. FPKM values calculated by Cufflinks were used as input for statistical analysis. The transcript quantification engine of Cufflinks (Cuffdiff) was used to calculate significant differences in transcript expression levels between groups [37]. P-values were corrected for multiple testing using the Benjamini–Hochberg method [38]. False discovery rate (FDR) adjusted p-values below 0.05 were considered as statistically significant evidence of gene differential expression between groups.

### Biological pathway analysis

Evidence of differential regulation of biological processes in the transcriptome datasets was tested using the goseq R Bioconductor package [39] which provides methods for performing analysis of Gene Ontology (GO) terms. FDR adjusted p-value of category enrichment were calculated and p-values below 0.05 were considered as statistically significant evidence of pathway differential regulation between groups.

## Results

### General features of gene transcription in the rat brain

We generated a high number of sequencing reads in the eight biological replicates of the four brain regions (53–116 M, 89.9 M on average), most of which were kept after trimming (97.8% on average) (Supplementary Table 1). Nearly 98% of the surviving reads could be aligned to the rat genome assembly (Rno6.0) leading to an exonic rate of 0.66 on average. Over 17,000 different genes were observed to be expressed in each sample (range 17,015–18,367). There were no significant differences in the total number of sequenced genes between brain regions.

(Supplementary Table 1). A total of 19,244 genes were observed to be expressed in at least one of the four brain regions (Supplementary Table 2).

Pairwise Pearson's correlation analysis of RNA sequencing data showed that correlations between samples were elevated (> 0.960). Correlation of sequencing data between biological replicates was greater within each brain region (0.981–0.988) than between different regions (0.960–0.976) (Supplementary Table 3).

### Transcriptome analysis identifies conserved gene transcription regulation pattern in the rat brain

Considering results from PCA and MDS, which suggest a lack of substantial differences in gene transcription within each brain region between gastrectomised and sham operated rats, we set out to define gene expression regulation shared in hypothalamus, hippocampus, brainstem and striatum or specific to one of these brain regions. We identified 2794 transcripts which were detected above the expression threshold but did not show evidence of statistically significant differential expression between brain regions (DESeq adjusted P ≥ 0.05) (Supplementary Table 4). Several of these transcripts were abundant and are known to play a role in brain function, including for example cystatin C (*Cst3*), the LDL receptor related protein 1 (*Lrp1*), which is downregulated in the brain of patients with Alzheimer's disease, the glucose-6-phosphate isomerase (*Gpi*), which promotes neuron survival, and the acyl-CoA synthetase long chain family member 3 (*Acsl3*), which encodes a protein involved in lipid biosynthesis and fatty acid degradation and known to be highly expressed in the brain.

### Brain regions show different patterns of gene transcription

**Fig. 1:**
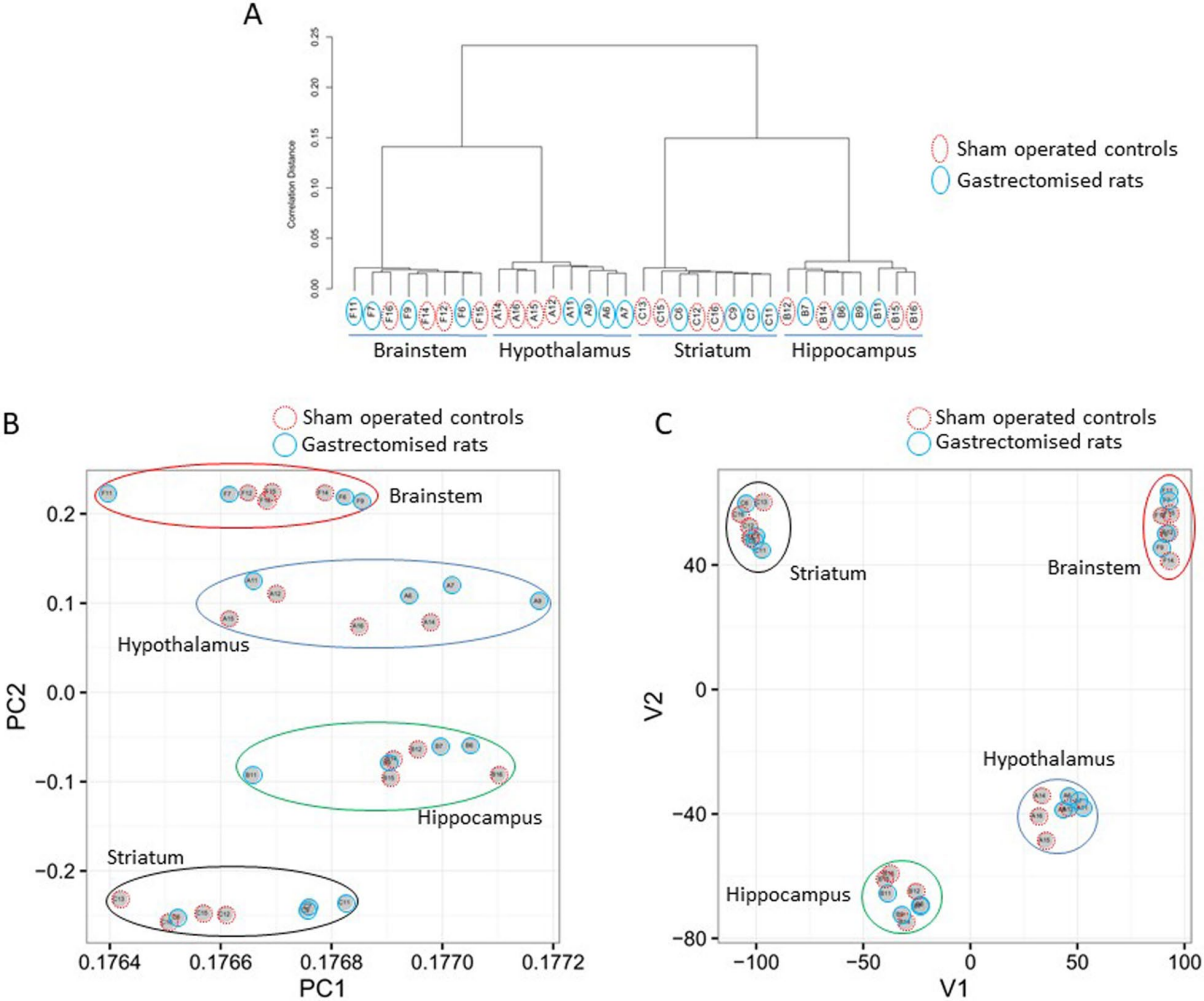
Illustration of regional patterns of transcription regulation in the rat brain. RNA sequencing data from four regions of the brain from the spontaneously diabetic Goto-Kakizaki rat were used. Clustering analysis of log2(RPKM) (**A**) illustrates the sequencing data consistency within brain regions, which were separated through principal component analysis (PCA) (**B**) and multi-dimensional scaling (MDS) (**C**). Individuals 6, 7, 9 and 11 were gastrectomised and individuals 12, 13, 14, 15 and 16 were sham operated. Circled letters refer to hypothalamus (**A**), hippocampus (**B**), striatum (**C**) and brainstem (**F**)

We next used the RNA sequencing data in hypothalamus, hippocampus, brainstem and striatum to identify differential gene expression patterns. Clustering analysis using the log2(RPKM) data (Fig. 1A) demonstrated strong similarities in sequencing data for each brain region, without separation of gastrectomised individuals and sham-operated controls. This was confirmed by PCA (Fig. 1B) and MDS (Fig. 1C), which also demonstrated the existence of specific patterns of gene transcription in each brain region.

**Fig. 2:**
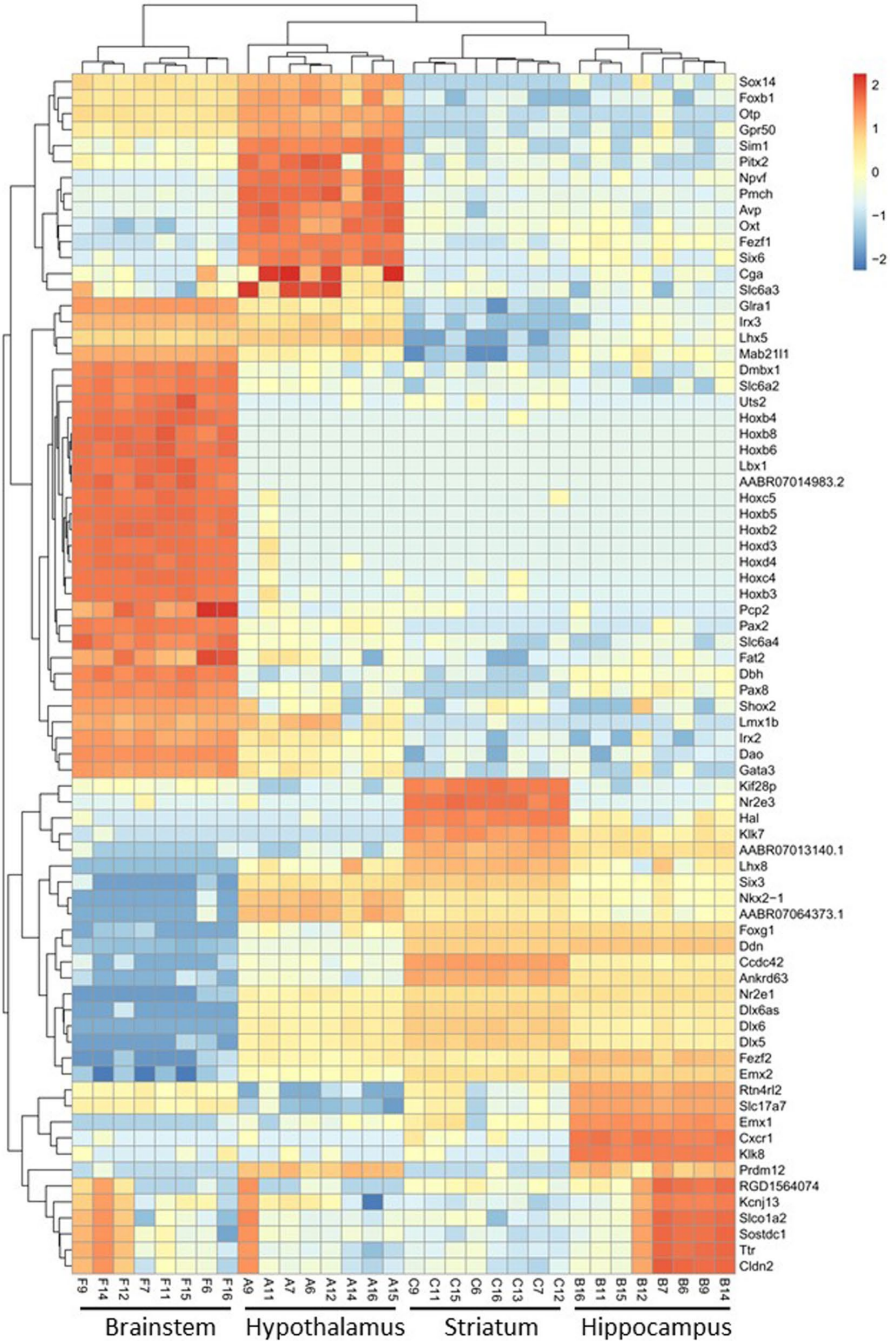
Hierarchical clustering of the transcriptome of brain regions based on log2(FPKM). Hypothalamus, hippocampus, brainstem and striatum of Goto-Kakizaki rats were dissected and used for RNA sequencing. Details of genes contributing to the separation of the transcriptomes derived in the four brain regions are given in Supplementary Table 5

Hierarchical clustering of the gene transcripts showing the most variable expression between regions based on the log2(FPKM) of cufflinks (Fig. 2) underlines their contribution to group separation and differences in transcriptional patterns between brain regions. Clustering identified overexpression of groups of genes that contribute the most to the separation of hypothalamus (e.g. *Avp*, *Cga*, *Fezf1*, *Foxb1*, *Gpr50*, *Npvf*, *Otp*, *Oxt*, *Pitx2*, *Pmch*, *Six6*, *Slc6a3*, *Sim1*, *Sox14*), hippocampus (e.g. *Cxcr1*, *Emx1*, *Klk8*, *Rtn4r2*, *Slc17a7*) or striatum (e.g. *Kif28p*, *Nr2e3*) to the other three brain regions. The most striking effect was the dominant contribution of elevated expression of twenty homeobox genes (*Dlx5*, *Dlx6*, *Dmbx1*, *Emx2*, *Hoxb2*, *b3*, *b4*, *b5*, *b6*, *b8*, *c4*, *c5*, *d3*, *d4*, *Lbx1*, *Lhx8*, *Lmx1b*, *Nkx2-1*, *Shox2*, *Six3*) (Fig. 2) to the separation of the brainstem transcriptome to that of the other three brain regions. Other contributors to this pattern of brainstem dominant overexpression include genes encoding the dopamine beta-hydroxylase (*Dbh*) and the neuromediator transporters *Slc6a2* and *Slc6a4*. Conversely, clustering identified pattern of specific downregulated gene transcription in the brainstem (e.g. *Ddn*, *Dlx5*, *Dlx6*, *Dlx6as*, *Emx2*, *Fezf2*, *Foxg1*, *Lhx8*, *Nr2e1*, *Six3*), the hypothalamus (*Slc17a7*) and the striatum (*Glra1*, *Irx3*, *Lhx5, Mab21l1*) (Fig. 2). These results suggest the existence of shared and distinct genomic regulations in the four brain regions that we investigated.

### Transcriptome analyses identify genes differentially expressed between brain regions

**Fig. 3:**
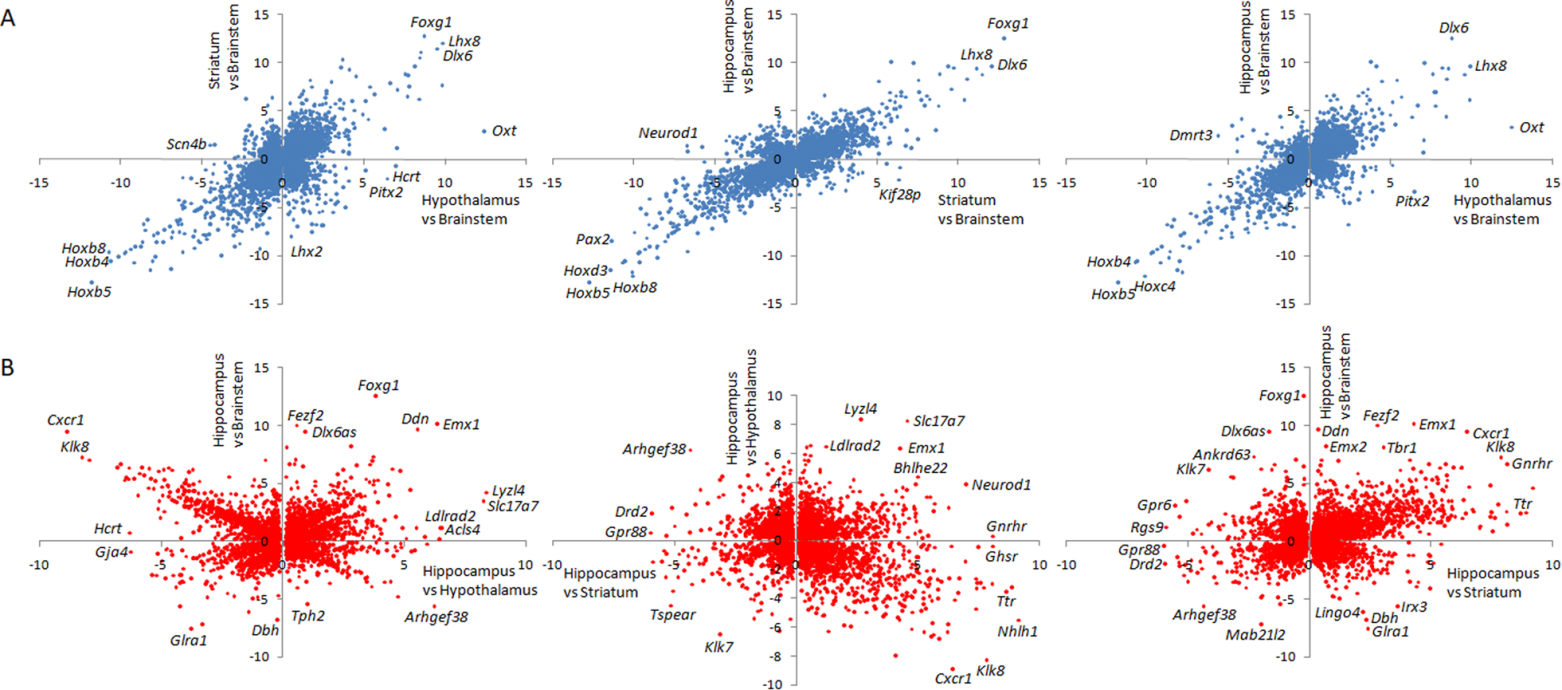
Illustration of conserved and discordant patterns of transcription regulation in brain regions. Data are shown for genes significantly differentially expressed in brainstem (**A**) and in hippocampus (**B**) when compared to the other three regions. Transcriptomes of hypothalamus, hippocampus, brainstem and striatum (n = 8 per region) were generated by RNA sequencing of samples from the Goto-Kakizaki rat strain. Statistical significance of differential expression between brain regions was determined by DESeq2 followed by adjustment for multiple testing. Differences in gene expression were statistically significant for DESeq adjusted P < 0.05. Details of differentially expressed genes in each pairwise comparison between brain regions are given in Supplementary Table 5

To analyse differential gene expression between brain regions, and identify region-specific differential transcription, pairwise comparisons of the hypothalamus, hippocampus, brainstem and striatum transcriptomes were carried out. A total of 16,602 genes showed evidence of statistically significant differential expression (DESeq adjusted P < 0.05) in at least one comparison between brain regions (Supplementary Table 5). Results from comparison analyses showed generally high levels of concordance (58–83%) in the genes differentially expressed between brain regions, even when conservation in the direction of gene expression changes was considered (40–71%) (Table 1). For example, differential expression of 83% genes between hippocampus and brainstem was replicated in the comparison between striatum and brainstem, even though direction of transcription changes was consistent for as much as 67% of the total number of differentially expressed genes, suggesting relatively high conservation of transcription regulation in brainstem when compared to other brain regions (Table 1, Fig. 3A). In contrast, patterns of up- and down-regulation of differentially expressed genes was more divergent when the hippocampus transcriptome was compared to that of the other brain regions (Fig. 3B). For example, only 40% of genes differential expressed between hippocampus and striatum were differentially regulated with consistent direction of expression between hippocampus and hypothalamus (Table 1, Fig. 3B).

**Table 1:** Pairwise comparisons of transcriptomes derived from distinct rat brain regions

	Transcriptome comparisons	DEG	Conserved direction of gene transcription change (% of total DEG)					
			Hippocampus vs Striatum	Hypothalamus vs Brainstem	Hypothalamus vs Hippocampus	Hypothalamus vs Striatum	Striatum vs Brainstem	Hippocampus vs Brainstem
Shared DEG	Hippocampus vs Striatum	10,384		3211 (31%)	4116 (40%)	6433 (62%)	6849 (66%)	4398 (42%)
	Hypothalamus vs Brainstem	10,866	6923 (64%)		4633 (43%)	4645 (43%)	7072 (65%)	7101 (65%)
	Hypothalamus vs Hippocampus	9630	6464 (67%)	6730 (70%)		6134 (64%)	2651 (28%)	6033 (63%)
	Hypothalamus vs Striatum	10,966	7503 (68%)	7421 (68%)	7247 (66%)		7786 (71%)	2690 (25%)
	Striatum vs Brainstem	13,156	8452 (64%)	8924 (68%)	7626 (58%)	9025 (69%)		8777 (67%)
	Hippocampus vs Brainstem	12,097	7744 (64%)	8505 (70%)	7481 (62%)	8075 (67%)	9990 (83%)	

Transcriptome data were generated by RNA sequencing of the same eight biological replicates in hippocampus, striatum, brainstem and hypothalamus of Goto-Kakizaki rats. Considering the lack of substantial differences in gene transcription between gastrectomised (n = 4) and sham operated (n = 4) rats, RNA sequencing data from the two experimental groups were pooled to carry out analyses of shared or specific gene expression in the brain regions. Significance of differentially expressed genes (DEG) was determined using DESeq2 followed by correction for multiple testing. Differences in gene expression were statistically significant for DESeq adjusted P < 0.05. Percentages of concordance between pairwise comparisons are calculated as the ratios between DEG and the total number of DEG

### Brain regions exhibit specific gene transcription regulation

**Fig. 4:**
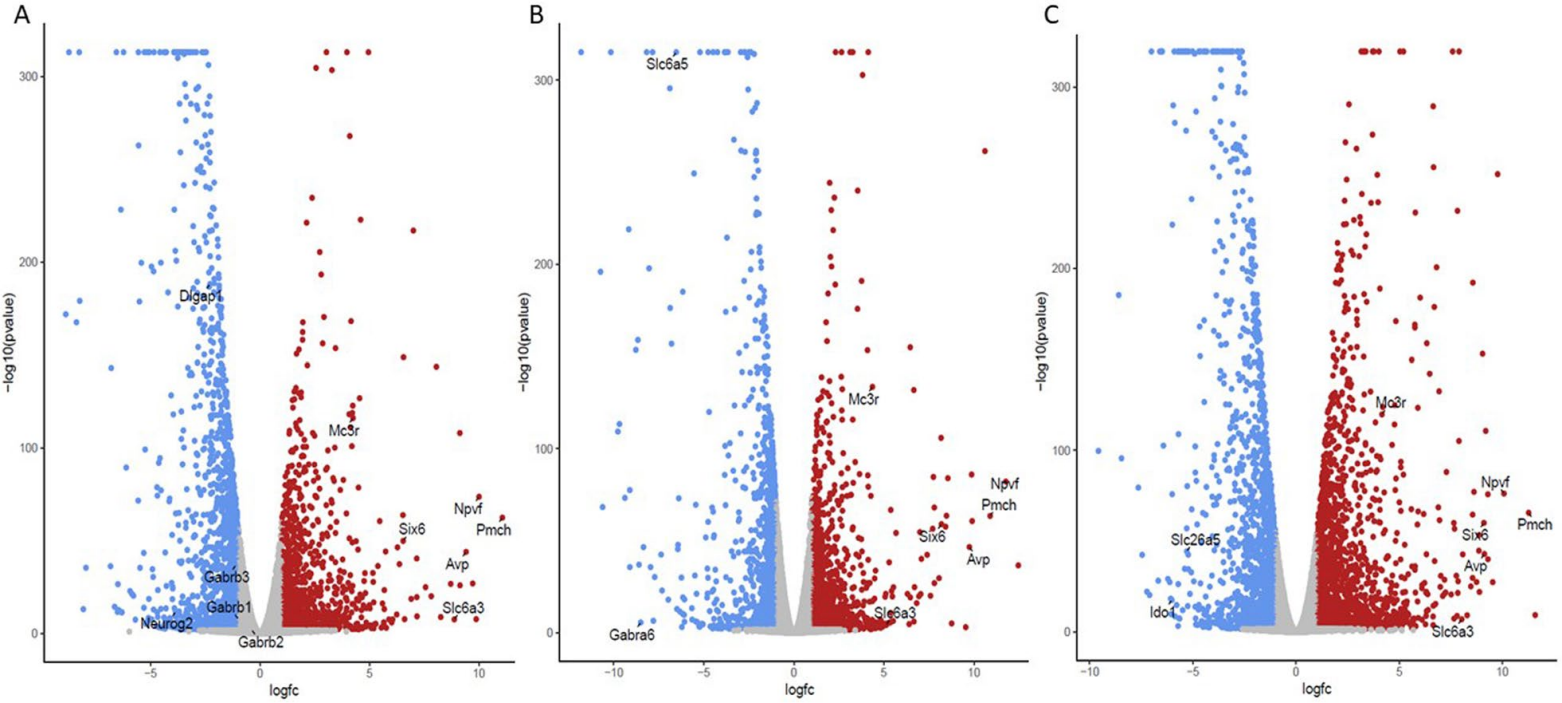
Illustration of hypothalamus-specific gene expression. Hypothalamus, hippocampus, brainstem and striatum of Goto-Kakizaki rats were dissected and used for RNA sequencing. Volcano plots were derived from pairwise comparisons of gene expression changes between hypothalamus and hippocampus (**A**), brainstem (**B**) and striatum (**C**) of Goto-Kakizaki rats using the Galaxy web platform (usegalaxy.org) [66]. Details of genes contributing to the separation of the transcriptomes derived in the hypothalamus and the other brain regions are given in Supplementary Table 5. Genes expressed specifically in the hypothalamus are listed in Supplementary Table 6

Pairwise analyses allowed us to identify genes significantly differentially expressed uniquely in hippocampus (n = 468), in striatum (n = 791), in hypothalamus (n = 477) and in brainstem (n = 1173) when compared to the other three regions (Supplementary Table 6). There was an excess of upregulated genes in hippocampus (n = 310; 66%) and in hypothalamus (n = 337; 71%). These results underline the strong and specific over-expression of genes encoding the arginine vasopressin (*Avp*) (LogFC > 9.13; DESeq adjusted P < 2.10 × 10^–42^), the melanocortin 3 receptor (*Mc3r*) (LogFC > 4.16; DESeq adjusted P < 6.20 × 10^–115^), the neuropeptide VF precursor (*Npvf*) (LogFC > 10.00; DESeq adjusted P < 9.00 × 10^–73^), the pro-melanin concentrating hormone (*Pmch*) (LogFC > 10.90; DESeq adjusted P < 1.30 × 10^–61^), the SIX homeobox 6 (*Six6*) (LogFC > 6.54; DESeq adjusted P < 3.70 × 10^–49^), and the solute carrier family 6 member 3 (*Slc6a3*) (LogFC > 5.12; DESeq adjusted P < 2.30 × 10^–4^) in the hypothalamus when compared to the other three brain regions (Fig. 4A–C). Analysis of the transcriptomes further highlights the massive downregulated transcription of above mentioned homeobox genes (LogFC = − 5.32 to − 12.7) and additional homeobox genes (*Evx2*, *Hoxa5*, *Hoxb1*, *Hoxb7*, *Hoxc6*, *Hoxd8*, *Irx4*, *Lhx4*, *Mnx1*, *Tlx1*, *Tlx3*) in this region when compared to the other three brain regions (Supplementary Table 6). The other brain regions also showed instances of specific gene expression, including for example the gamma-aminobutyric acid type A receptor subunits β1 (*Gabrb1*) (LogFC > 0.10; DESeq adjusted P < 1.20 × 10^–7^), β2 (*Gabrb2*) (LogFC > 3.22; DESeq adjusted P < 7.20 × 10^–27^) and β3 (*Gabrb3*) (LogFC > 3.42; DESeq adjusted P < 3.90 × 10^–12^), the neurogenin 2 (*Neurog2*) (LogFC > 3.86; DESeq adjusted P < 2.00 × 10^–11^) and the DLG associated protein 1 (*Dlgap1*) (LogFC > 2.19; DESeq adjusted P < 2.90 × 10^–174^) in the hippocampus, the indoleamine 2,3-dioxygenase 1 (*Ido1*) (LogFC > 5.29; DESeq adjusted P < 2.20 × 10^–17^) and the solute carrier family 26, member 5 (*Slc26a5*) (LogFC > 4.74; DESeq adjusted P < 7.00 × 10^–44^) in the striatum and the gamma-aminobutyric acid type A receptor subunit α6 (*Gabra6*) (LogFC > 6.96; DESeq adjusted P < 9.50 × 10^–5^) and the solute carrier family 6 member 5 (*Slc6a5*) (LogFC > 6.29; DESeq adjusted P < 4.50 × 10^–306^) in the brainstem (Supplementary Table 6).

### Brain regions exhibit shared and specific biological pathways

Using the rat brain transcriptomes that we generated, we identified 45 biological processes differentially regulated in at least one pairwise comparison between brain regions (Table 2). As expected, over 37% of the differentially represented GO terms are related to neuronal function (e.g. synapse, axon, dendrite), whereas the remaining are relevant to general cell components (e.g. cytoplasm, membrane, Golgi apparatus, nucleoplasm) and physiological and molecular mechanisms (e.g. phosphorylation, calcium channel complex, GTPase activity, calmodulin binding, ion channel binding). Systematic occurrence of the process “protein binding” (GO ID: 0005515) in all six pairwise comparisons indicates that different subsets of genes in the pathway contribute to the enrichment of the pathway. Along the same line, GO processes differentially regulated in more than four pairwise comparisons between brain regions may involve a combination of site-specific and overlapping series of genes as exemplified by the processes postsynaptic membrane (GO:0045211) and dendritic spine (GO:0043197) (Supplementary Table 7). In contrast, as many as 26 processes were specific to a single pairwise comparison, the majority of which [22] underlies differences in GO terms between the hippocampus transcriptome and that of another brain region. Genes contributing to several biological functions specific to the hippocampus are illustrated in Supplementary Table 8 with the GO terms terminal bouton (GO:0043195), which covers structures and mechanisms occurring at the extremity of the axon involved in the release of neurotransmitters, and is therefore directly relevant to neuronal function, and cell adhesion (GO:007156) and cellular response to starvation (GO:009267), which may provide novel insights into hippocampus-specific transcriptional regulation.

**Table 2:** Biological pathway analysis of rat brain transcriptomes

GO:ID	GO Term	*N* Genes	Hypothalamus vs Striatum	Hippocampus vs Striatum	Striatum vs Brainstem	Hypothalamus vs Hippocampus	Hypothalamus vs Brainstem	Hippocampus vs Brainstem
0005509	Calcium ion binding	721	–	–	–	9.9 (5.2 × 10^–5^)	–	–
0005515	Protein binding	7830	44.4 (5.1 × 10^–20^)	30.9 (3.9 × 10^–14^)	42.8 (2.6 × 10^–19^)	38.8 (1.5 × 10^–17^)	41.3 (8.3 × 10^–19^)	37.5 (5.3 × 10^–17^)
0005516	Calmodulin binding	189	–	–	–	–	–	10.3 (3.5 × 10^–5^)
0005654	Nucleoplasm	3542	–	–	–	–	10.2 (3.8 × 10^–5^)	–
0005737	Cytoplasm	11,393	16.6 (6.1 × 10^–8^)	–	16.5 (7.1 × 10^–8^)	18.6 (8.3 × 10^–9^)	19.6 (3.0 × 10^–9^)	–
0005743	Mitochondrial inner membrane	417	–	–	–	–	–	11.6 (8.9 × 10^–6^)
0005783	Endoplasmic reticulum	1777	–	–	–	8.6 (1.9 × 10^–4^)	–	–
0005794	Golgi apparatus	1457	–	–	–	–	11.0 (1.6 × 10^–5^)	–
0005829	Cytosol	4149	–	–	–	9.8 (5.7 × 10^–5^)	–	10.9 (2.0 × 10^–5^)
0005856	Cytoskeleton	2234	–	–	–	11.4 (1.1 × 10^–5^)	–	–
0005886	Plasma membrane	6368	18.9 (6.0 × 10^–9^)	29.8 (1.2 × 10^–13^)	–	14.7 (4.1 × 10^–7^)	10.9 (1.9 × 10^–5^)	22.8 (1.2 × 10^–10^)
0005887	Integral component of plasma membrane	1370	–	16.3 (8.7 × 10^–8^)	–	–	–	–
0005911	Cell–cell junction	532	–	9.1 (1.2 × 10^–4^)	–	–	–	–
0006887	Exocytosis	423	–	–	–	9.7 (6.4 × 10^–5^)	–	–
0007156	Homophilic cell adhesion	161	–	–	–	9.5 (7.7 × 10^–5^)	–	–
0007268	Synaptic transmission	879	–	13.0 (2.2 × 10^–6^)	–	–	–	–
0007399	Nervous system development	2458	11.3 (1.3 × 10^–5^)	–	–	–	–	–
0007411	Axon guidance	258		–	–	9.6 (6.6 × 10^–5^)	–	9.6 (6.5 × 10^–5^)
0007420	Brain development	966	–	–	–	8.8 (1.6 × 10^–4^)	–	–
0009267	Cellular response to starvation	196	–	9.0 (1.3 × 10^–4^)	–	–	–	–
0010976	Positive regulation of neuron projection development	235	–	10.7 (2.4 × 10^–5^)	–	–	–	–
0014069	Postsynaptic density	438	10.4 (3.0 × 10^–5^)	–	–	11.4 (1.2 × 10^–5^)	–	–
0016020	Membrane	10,363	–	10.4 (3.0 × 10^–5^)	–	–	–	–
0016310	Phosphorylation	2210	–	–	13.3 (1.7 × 10^–6^)	–	–	–
0030054	Cell junction	2203	13.6 (1.3 × 10^–6^)	11.2 (1.4 × 10^–5^)	–	16.6 (6.2 × 10^–8^)	–	–
0030424	Axon	848	11.5 (1.0 × 10^–5^)	12.8 (2.8 × 10^–6^)	–	9.5 (7.2 × 10^–5^)	9.8 (5.4 × 10^–5^)	12.8 (2.7 × 10^–6^)
0030425	Dendrite	818	9.8 (5.3 × 10^–5^)	10.5 (2.6 × 10^–5^)	–	13.4 (1.6 × 10^–6^)	12.8 (2.7 × 10^–6^)	15.9 (1.2 × 10^–7^)
0031175	Neuron projection development	1109	–	9.4 (8.1 × 10^–5^)	–	–	–	–
0032870	Cell response to hormone stimulus	693	–	–	–	9.1 (1.1 × 10^–4^)	–	–
0034704	Calcium channel complex	76	–	–	–	9.4 (8.7 × 10^–5^)	–	–
0035690	Cellular response to drug	110	–	–	–	8.8 (1.6 × 10^–4^)	–	–
0043005	Neuron projection	1666	12.3 (4.6 × 10^–6^)	14.1 (7.3 × 10^–7^)	–	8.9 (1.4 × 10^–4^)	–	–
0043025	Neuronal cell body	763	15.4 (2.0 × 10^–7^)	9.5 (7.2 × 10^–5^)	–	15.3 (2.2 × 10^–7^)	13.6 (1.2 × 10^–6^)	9.9 (5.2 × 10^–5^)
0043195	Terminal bouton	139	–	–	–	9.1 (1.1 × 10^–4^)	–	–
0043197	Dendritic spine	237	12.6 (3.3 × 10^–6^)	10.7 (2.3 × 10^–5^)	12.4 (4.1 × 10^–6^)	13.5 (1.4 × 10^–6^)	–	–
0043209	Myelin sheath	80	–	14.8 (3.8 × 10^–7^)	16.7 (5.6 × 10^–8^)	–	25.3 (9.9 × 10^–12^)	22.6 (1.5 × 10^–10^)
0043547	Positive regulation of GTPase activity	382	–	–	–	9.3 (8.8 × 10^–5^)	–	–
0043565	Sequence-specific DNA binding	1589	–	–	–	9.4 (8.3 × 10^–5^)	–	13.3 (1.7 × 10^–6^)
0044325	Ion channel binding	160	–	10.1 (4.0 × 10^–5^)	–	–	–	–
0045202	Synapse	1597	19.5 (3.4 × 10^–9^)	13.1 (2.1 × 10^–6^)	9.9 (4.9 × 10^–5^)	21.4 (5.4 × 10^–10^)	–	14.2 (7.1 × 10^–7^)
0045211	Postsynaptic membrane	336	11.0 (1.6 × 10^–5^)	12.0 (6.1 × 10^–6^)	10.7 (2.3 × 10^–5^)	15.6 (1.6 × 10^–7^)	–	–
0048471	Perinuclear region of cytoplasm	775	–	12.5 (3.8 × 10^–6^)	–	11.2 (1.4 × 10^–5^)	–	–
0060291	Long-term synaptic potentiation	215	–	10.3 (3.2 × 10^–5^)	–	10.7 (2.2 × 10^–5^)	–	–
0070062	Extracellular exosome	110	–	21.2 (6.5 × 10^–10^)	10.5 (2.8 × 10^–5^)	13.9 (9.0 × 10^–7^)	–	12.4 (4.1 × 10^–6^)
0097481	Neuronal postsynaptic density	8	–	–	–	9.8 (5.5 × 10^–5^)	–	–

Transcriptome data were generated by RNA sequencing of the same eight biological replicates in hippocampus, striatum, brainstem and hypothalamus of Goto-Kakizaki rats. Considering the lack of substantial differences in gene transcription between gastrectomised (n = 4) and sham operated (n = 4) rats, RNA sequencing data from the two experimental groups were pooled to carry out analyses of shared or specific gene expression in the brain regions. Sequence reads were aligned to the rat genome assembly Rno6.0. Evidence of differential regulation of biological processes between the transcriptome datasets was tested using the goseq R Bioconductor package. Gene Ontology (GO) terms and references, as well as the number of rat genes represented in the GO:ID, are given. False discovery rate (FDR) adjusted p-values of category enrichment were calculated. A FDR < 0.1 of filtered p-values was considered as statistically significant evidence of pathway differential regulation between groups. Normalised Enrichment Scores (NES) and adjusted p-values (in parentheses) are given for each pairwise comparison between brain regions for pathways showing evidence of statistically significant enrichment

### Brain transcriptomes suggest an impact of bariatric surgery on hypothalamus vascularisation and angiogenesis

To test the molecular consequences of VSG and subsequent normalisation of glycemic control on brain gene expression, we analysed changes in gene expression in striatum, brainstem, hypothalamus and hippocampus between gastrectomised and sham-operated GK rats. No differences in gene transcription were detected between the two rat groups in striatum, brainstem and hippocampus. Evidence of statistically significant differential expression between VSG and sham operated rats (DESeq adjusted P < 0.05) was obtained for only 41 genes in hypothalamus (Table 3). All transcripts but one (*Lrp1b*) were upregulated in gastrectomised rats. Strongest statistical significance (DESeq adjusted P < 10^–4^) was obtained with the transcripts encoding the collagen type VIII alpha 1 chain (*Col8a1*), the scavenger receptor class A, member 5 (*Scara5*), the solute carrier family 13, member 4 (*Slc13a4*) and schlafen 5 (*Slfn5*). The strongest expression ratio between VSG and control was obtained with the transcripts encoding the hydrocarboxylic acid receptor 1 (*Hcar1, Gpr81*) (Log FC = 4.303, DESeq adjusted P = 0.04) and the sulfate transporter *Slc13a4* (Log FC = 2.543, DESeq adjusted P = 4.7 × 10^–5^).

**Table 3:** Details of genes differentially expressed in hypothalamus between gastrectomised and sham operated Goto-Kakizaki rats

Acronym	Gene description	log_FC	DESeq P	DESeq adjusted P	log_CPM	EdgeR P	EdgeR adjusted P
AABR07067267.1	–	1.172	1.3 × 10^–5^	0.011	1.258	4.3 × 10^–5^	0.010
Adamtsl4	ADAMTS-like 4	0.815	2.6 × 10^–7^	8.1 × 10^–4^	2.887	7.5 × 10^–5^	0.013
Aifm3	Apoptosis inducing factor, mitochondria associated 3	0.442	3.3 × 10^–6^	0.005	6.326	1.9 × 10^–5^	0.006
Akr1c19	Aldo–keto reductase family 1, member C19	0.809	6.2 × 10^–5^	0.035	2.145	4.3 × 10^–4^	0.042
Apol3	Apolipoprotein L3	0.947	2.9 × 10^–6^	0.005	2.277	1.0 × 10^–5^	0.004
Asap3	ArfGAP, SH3 domain, ankyrin repeat and PH domain 3	0.775	3.8 × 10^–5^	0.025	2.332	3.8 × 10^–4^	0.040
Bmp7	Bone morphogenetic protein 7	1.941	1.1 × 10^–4^	0.049	2.228	4.4 × 10^–4^	0.043
C4b	Complement C4B	1.476	1.3 × 10^–7^	4.8 × 10^–4^	1.606	9.0 × 10^–9^	3.4 × 10^–5^
Cald1	Caldesmon 1	0.834	3.5 × 10^–5^	0.025	5.844	7.7 × 10^–9^	3.4 × 10^–5^
Col27a1	Collagen type XXVII alpha 1 chain	0.509	7.0 × 10^–5^	0.037	5.308	1.4 × 10^–4^	0.019
Col8a1	Collagen type VIII alpha 1 chain	1.366	4.1 × 10^–9^	3.8 × 10^–5^	1.937	2.3 × 10^–6^	0.002
Col8a2	Collagen type VIII alpha 2 chain	1.789	4.3 × 10^–6^	0.005	3.282	3.2 × 10^–7^	3.5 × 10^–4^
Ctsc	Cathepsin C	0.808	8.3 × 10^–7^	0.002	3.108	8.2 × 10^–5^	0.014
Dock6	Dedicator of cytokinesis 6	0.557	8.7 × 10^–5^	0.043	4.039	3.0 × 10^–4^	0.035
Eng	Endoglin	0.508	1.2 × 10^–5^	0.011	4.598	8.6 × 10^–5^	0.014
Hcar1 (Gpr81)	Hydrocarboxylic acid receptor 1	4.303	7.8 × 10^–5^	0.040	−0.023	3.0 × 10^–7^	3.5 × 10^–4^
Hspg2	Heparan sulfate proteoglycan 2	1.233	1.9 × 10^–5^	0.015	1.906	8.6 × 10^–7^	7.4 × 10^–4^
Il1r1	Interleukin 1 receptor type 1	0.771	5.1 × 10^–7^	0.001	3.148	2.2 × 10^–5^	0.007
Itpr3	Inositol 1,4,5-trisphosphate receptor type 3	0.675	4.5 × 10^–5^	0.027	2.946	7.0 × 10^–4^	0.059
Lamb2	Laminin subunit beta 2	0.654	4.8 × 10^–5^	0.028	6.142	1.2 × 10^–6^	9.7 × 10^–4^
LOC100363469	Ribosomal protein S24-like	1.549	1.2 × 10^–5^	0.011	0.486	3.6 × 10^–7^	3.8 × 10^–4^
LOC689130	Ferritin heavy chain 1	0.876	6.6 × 10^–5^	0.036	2.433	7.5 × 10^–4^	0.060
Lrp1b	LDL receptor related protein 1B	−1.221	4.5 × 10^–5^	0.027	2.386	0.0025	0.120
Mmrn2	Multimerin 2	0.734	3.8 × 10^–6^	0.005	3.060	3.0 × 10^–5^	0.008
Mrvi1	Murine retrovirus integration site 1 homolog	1.605	2.4 × 10^–6^	0.004	2.406	1.8 × 10^–8^	4.9 × 10^–5^
Nexn	Nexin	0.872	1.0 × 10^–6^	0.002	2.565	1.3 × 10^–4^	0.019
Pdgfrb	Platelet derived growth factor receptor beta	0.728	8.2 × 10^–5^	0.041	4.723	2.1 × 10^–6^	0.002
Pear1	Platelet endothelial aggregation receptor 1	1.125	1.0 × 10^–4^	0.047	3.461	1.2 × 10^–7^	1.8 × 10^–4^
Plekhg2	pleckstrin homology and RhoGEF domain containing G2	0.642	3.6 × 10^–5^	0.025	3.167	5.4 × 10^–4^	0.048
Ppp1r3b	Protein phosphatase 1 regulatory subunit 3B	0.778	3.9 × 10^–5^	0.025	2.434	6.9 × 10^–4^	0.059
Prodh	Proline dehydrogenase 1	0.395	1.1 × 10^–5^	0.011	6.365	6.2 × 10^–5^	0.012
Rcsd1	RCSD domain containing 1	0.778	1.8 × 10^–5^	0.015	3.008	9.8 × 10^–5^	0.016
Scara5	Scavenger receptor class A, member 5	1.866	1.0 × 10^–8^	4.8 × 10^–5^	0.812	1.2 × 10^–10^	1.1 × 10^–6^
Slc13a4	Solute carrier family 13, member 4	2.543	7.5 × 10^–9^	4.7 × 10^–5^	4.126	8.3 × 10^–6^	0.004
Slc16a12	Solute carrier family 16, member 12	0.609	9.2 × 10^–5^	0.044	3.284	3.9 × 10^–4^	0.040
Slc2a12	Solute carrier family 2, member 12	1.031	6.3 × 10^–6^	0.007	3.709	2.2 × 10^–6^	0.002
Slfn5	Schlafen 5	0.694	2.0 × 10^–9^	3.7 × 10^–5^	4.955	3.5 × 10^–8^	7.4 × 10^–5^
Stat6	Signal transducer and activator of transcription 6	0.585	1.9 × 10^–5^	0.015	3.722	1.2 × 10^–4^	0.018
Tgm2	Transglutaminase 2	1.168	7.2 × 10^–6^	0.008	4.109	5.7 × 10^–10^	3.6 × 10^–6^
Thbs2	Thrombospondin 2	1.502	1.7 × 10^–6^	0.003	3.096	9.7 × 10^–8^	1.7 × 10^–4^
Tns2	Tensin 2	0.499	1.4 × 10^–5^	0.011	5.596	6.0 × 10^–6^	0.003

Gene transcription was analysed by RNA sequencing of hypothalamus samples from Goto-Kakizaki rats 136 days after vertical sleeve gastrectomy (VSG) (n = 4) or sham operation (n = 4). Sequencing reads were aligned to the rat genome reference RNO6.0. Differential expression analyses between VSG and sham operated rats were performed using DESeq2 and EdgeR and corrected for multiple testing. *FC* Fold Change, *CMP* Counts-Per-Million Reads

Even though pathway analysis of the hypothalamus transcriptomes in VSG and sham operated rats failed to identify statistical differences in the regulation of biological functions, inspection of genes differentially expressed between the two rat groups identified predominant representation of genes involved in the control of the vascular extracellular matrix (ECM) (*Adamtsl4*, *Bmp7*, *Eng*, *Hspg2*, *Mmrn2*, *Thbs2*), the endothelium (*Apol3*, *Cald1*, *Col8a1*, *Col8a2*, *Eng*, *Hcar1/Gpr81*, *Hspg2*, *Lamb2*, *Pear1*), cell migration (*Asap3*, *Dock6*, *Nexn*, *Pdgfrb*, *Tns2*) and more generally angiogenesis (*Adamts*, *Apol3*, *Cald1*, *Col8a1*, *Col8a2*, *Eng*, *Hcar1/Gpr81*, *Lamb2*, *Mmrn2, Pear1*, *Scara5*, *Thbs2*) (Table 3). These findings suggest that bariatric surgery and subsequent improvement in glucose homeostasis in a rodent model of T2D devoid of obesity do not result in strong alteration in neuronal transcriptional regulations in the brain but instead lead to changes in the hypothalamus vascularisation and angiogenesis.

## Discussion

We report detailed transcriptome atlases of four brain regions in a rat model of spontaneously-occurring lean T2D, which were applied to identify gene expression changes accompanying diabetes remission following bariatric surgery. We identified series of genes consistently expressed across all brain regions, as well as genes differentially expressed between regions and a subset of genes specifically expressed in a single region. Gastrectomy-promoted diabetes remission, which occurs in the absence of dramatic changes in the brain transcriptome, involves altered hypothalamic expression of several genes involved in the regulation of the ECM and the endothelium.

Transcriptome profiling provides opportunities to advance knowledge in the spatio-temporal regulation of brain gene expression that adapts to environmental influences, and to identify functional alterations associated with neurodegenerative and neurobehavioural conditions, as well as cardiometabolic diseases [40, 41]. Our RNA sequencing-based brain transcriptome atlas generates comprehensive information on the level of expression of genes in the hypothalamus, hippocampus, brainstem, and striatum. Several genes showing the highest level of expression in the four brain regions encode proteins involved in neuron structure and function, including the myelin basic protein (*Mbp*), the microtubule associated proteins 1B (*Map1b*) and 2 (*Map2*), and the stearoyl-Coenzyme A desaturase 2 (*Scd2*), and in the etiopathogenesis of Alzheimer's disease (*Apoe*) [42]. In addition, consistent high level of transcripts of six tyrosine 3-monooxygenases (*Ywhab*, *Ywhae*, *Ywhag*, *Ywhah*, *Ywhaq*, *Ywhaz*) in the four brain regions underlines essential biochemical activities of signal transduction pathways and catecholamine biosynthesis [43]. Furthermore, the presence of unannotated transcripts at high level in the four brain regions (eg. LOC691995, RGD1312005) deepens the brain gene expression atlas with proteins of as yet unknown biological function.

Comparative analyses of the transcriptome data identified biological pathways enriched in brainstem, hippocampus, hypothalamus and striatum or shared between brain regions. In many instances the GO terms that were differentially enriched between brain regions underline neuron function (e.g. axon guidance) and often point to closely related neuronal structural features involving overlapping gene sets (e.g. Postsynaptic density GO:0014069 and postsynaptic membrane GO:0045211, neuron projection development GO:0031175 and neuron projection GO:0043005). We also noted differentially enriched GO terms that relate to signalling mechanisms (e.g. Calcium ion binding, regulation of GTPase activity, ion channel binding), which may also involve region-specific expression of genes.

Differential gene expression data in our experimental conditions suggest that the known functional and structural features of brain structures [44, 45] arise despite the very small proportion of genes specifically overexpressed in a single brain region (2.5–6.4%). Several transcripts known to play a role in brain function or in chronic diseases and predominantly or exclusively detected in the hypothalamus include the arginine vasopressin (*Avp*), which generates the neuropeptide hormone arginine vasopressin, the neurophysin 2 and copeptin, and the oxytocin/neurophysin I prepropeptide (*Oxt*), which generates the oxytocin and neurophysin I, the SIM bHLH transcription factor 1 (*Sim1*), which is associated with abnormalities of brain development, the neuropeptide VF precursor (*Npvf*) and the dopamine transporter *Slc6a3*. Of note, genes involved in melanocortin regulation and melanocyte function (*Pomc*, *Mc3r, Pmch*, *Gpr50*), which are involved in susceptibility to obesity and other disorders [46, 47], are strongly expressed in the hypothalamus. Conversely, transcripts encoding the glutamate transporter *Slc17a7*, which is present in membranes of synaptic vesicles and is expressed in neuron-rich regions of the brain, were present at very low level specifically in the hypothalamus.

The other brain regions also showed instances of overexpression of specific transcripts which may underlie enrichment in GO terms and contribute to their specific structural and functional features. For example, transcripts encoding the cadherin *Fat2*, which plays an important role in cerebellum development, the amino acid oxidase *Dao*, which may be involved in schizophrenia, the tryptophan hydroxylase 2 (*Tph2*), which is responsible for the biosynthesis of serotonin, the dopamine β-hydroxylase (*Dbh*), which converts dopamine to norepinephrine, the inhibitory neurotransmitter *Gabra6* and the neurotransmitter transporters *Slc6a2*, *Slc6a4* and *Slc6a5* were expressed predominantly in the brainstem. The genes encoding neuronal differentiation 6 (*Neurod6*) and basic helix-loop-helix family member e22 (*Bhlhe22*), which are involved in neuron differentiation were strongly expressed in the hippocampus. Conversely, transcripts encoding the LIM homeobox 8 (*Lhx8*), which is involved in neuronal differentiation, the distal-less homeobox 1(*Dlx1*), which may regulate inhibitory neurons in the brain, and the forkhead box G1 (*Foxg1*), which is responsible for neurodevelopmental disorders, were absent in the brainstem. Transcripts encoding the proteins *Slc17a6* and *Slc18a2*, which are located in synaptic vesicles for the transport of neurotransmitters, were not expressed in the striatum.

Dysfunctions in the central nervous system contribute to the etiopathogenesis of cardiometabolic diseases [3]. Knowledge of molecular mechanisms involved in the gut-brain axis has led to improved therapeutic solutions for these diseases [48]. Our repository of brain RNA sequencing data provide information on region-specific transcript abundance of risk genes identified through GWAS for these diseases. Using a list of candidate genes previously used to annotate risk genes expressed in the mouse brain [49], we show predominant expression of candidate genes for obesity in hypothalamus (*Asb4*, *Calcr*, *Cbln1*, *Pomc*, *Sim1*), hippocampus (*Bdnf*, *Grp*), striatum (*Cep295*, *Rarb*) or in brainstem (*Fam57b*, *Gprc5b*, *Tfap2b*) (Supplementary Table 9). Transcripts for several candidate genes for obesity were absent in the whole brain or absent in specific regions of the brain (*Cyp17a1*, *Gdf15*, *Gpr151*, *Lmx1b*, *Olig3*, *Sbk1*, *Sim1*, *Skor1*, *Tnni3k*). Similarly, transcripts of candidate genes for type 2 diabetes (*Blk*, *C2cd4a*, *C2cd4b*, *Cdkn2a*, *Hnf1a*, *Mtnr1b*, *Pou5f1*, *Slc30a8*) and type 1 diabetes (*Cd69*, *Il2*, *IL27*) were not expressed in the brain. Our brain transcription repository can also be used to assess the relevance of risk genes for other diseases based on transcript abundance. For example, we identified transcripts of candidate genes for autism not expressed in the brain (*Pax5*) or specifically absent in brainstem (*Arx*, *Foxg2*) or striatum (*Ebf3*) (Supplementary Table 9).

Our brain RNA sequencing data allowed us to investigate the effects of disrupted brain-gut connections and gastrectomy-promoted improvement of glucose homeostasis on brain function. Increasing evidence supports the adverse impact of obesity and diabetes on brain structure and cognitive functioning, which are at least partly reversed by bariatric surgery through mechanisms that may involve gut hormones, inflammatory cytokines and remyelination [50]. Gastrectomy-promoted improvement in glycemic control in lean GK rats is a dynamic process characterized by transient weight loss and permanent reduction of hyperglycemia [22], which may induce stage-dependent changes in brain transcription regulation. Our transcriptome data provide molecular information in a situation where body weight has returned to that of sham-operated GK rats whilst sustained reduction of hyperglycemia is achieved. Gene expression changes between VSG and sham operated GK rats were restricted to the hypothalamus and pointed to changes in the brain-blood barrier, which is modified in diabetes [51]. They may not be relevant to gastrectomy promoted resolution of obesity but instead underlie molecular adaptations to VSG in the context of spontaneously occurring T2D in the absence of obesity, which is caused in the GK strain by naturally occurring genetic polymorphisms [52, 53].

**Fig. 5:**
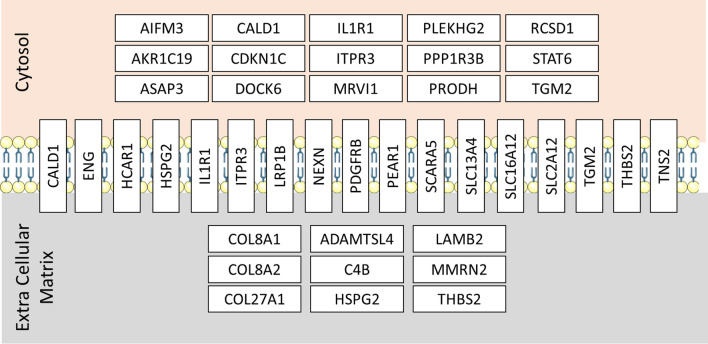
Schematic localisation of proteins encoded by genes differentially expressed in the hypothalamus between gastrectomized and sham-operated Goto-Kakizaki rats. Data are from the Human Proteome Atlas (www.proteinatlas.org) and the GeneCards database (www.genecards.org). Details of the genes and the statistics of differential transcription are given in Table 3

Annotations of genes significantly overexpressed in the hypothalamus of VSG rats show that the vast majority is involved in the structure and function of the vascular endothelium and the regulation of cell differentiation and migration, the ECM and, most prominently, angiogenesis (Fig. 5). Reports of altered cerebrovascular remodelling, endotheliun dysfunction and collagen deposition in the middle cerebral artery of the GK strain [54, 55] underline the pathophysiological relevance of gene transcription changes identified in the hypothalamus of VSG GK rats. Elevated expression of the RNA predicted to encode the ferritin heavy chain 1 (LOC689130) in VSG GK rats also suggests structural changes in the endothelial glycocalyx [56]. The gene showing the greatest stimulation of transcription in VSG rats (*Hcar1*, *Gpr81*), which encodes a protein activated by lactate and involved in enhanced brain angiogenesis, stimulated cerebral VEGFA and ghrelin secretion, may explain VSG-promoted improvement of glucose homeostasis in gastrectomised GK rats [57–59]. Increased activity in VSG GK rats during the dark phase (bioRxiv 2023.10.09.561476) may result in a rise in lactate production by skeletal muscle, which may in turn stimulate HCAR1 activity.

Of relevance to changes in the regulation of the brain-blood barrier, VSG GK rats exhibit significant upregulated expression of the type 1 membrane glycoprotein endoglin and the bone morphogenetic protein 7, which both contribute through distinct mechanisms to the maintenance of the ECM [60], suggesting improved vascular homeostasis and maintenance of endothelium morphology [61]. Increased expression of type 8 collagen, which is known to be enhanced by TGFβ1 [62] (Log FC = 0.441, DESeq P = 4.2 × 10^–3^; DESeq adjusted P = 0.3) may contribute, with overexpressed TGFβ1 coreceptors and interacting partners bone morphogenetic protein 7 (*Bmp7*), thrombospondin 2 (*Thbs2*) and endoglin [60, 63], to the regulation of the ECM and vascular function in the hypothalamus of VSG GK rats.

In conclusion, results from comprehensive brain transcriptome analysis provide a detailed atlas of regional gene expression in the diabetic rat brain. Our transcriptome data suggest that diabetes remission following gastrectomy in a model of spontaneously-occurring diabetes devoid of obesity does not affect brain neuronal regulation but induces functional changes in the hypothalamus dominated by altered control of the endothelium and the ECM. Further biochemical and physiological analyses are required to confirm our observation and provide deeper characterisation of the biological mechanisms involved. These mechanisms may be relevant to the emerging concept of hypothalamic connectivity associated with bariatric surgery in humans [64, 65].

## Supplementary Information

Supplementary Material 1

Supplementary Material 2

Supplementary Material 3

Supplementary Material 4

Supplementary Material 5

Supplementary Material 6

Supplementary Material 7

Supplementary Material 8

Supplementary Material 9

## Data Availability

All data generated and/or analysed during this study are included in this published article and its supplementary information file.

## Abbreviations

CNS: Central nervous system
ECM: Extracellular matrix
FDR: False discovery rate
GK: Goto-Kakizaki
GLP-1: Glucagon-like peptide 1
GO: Gene Ontology
MDS: Multi-dimensional scaling
PCA: Principal component analysis
T2D: Type 2 diabetes
VSG: Vertical sleeve gastrectomy
