# Supplementary material for: Transcriptome atlases of rat brain regions and their adaptation to diabetes resolution following gastrectomy in the Goto-Kakizaki rat
Source: Mol Brain. 2025 Feb 7;18:9. doi: 10.1186/s13041-025-01176-z (PMC11806591; doi:10.1186/s13041-025-01176-z)
Supplement: Supplementary file 1 — Supplementary Material 1 [file 13041_2025_1176_MOESM1_ESM.docx]

**Supplementary Table 1. Summary statistics of RNA sequencing of eight samples from each of the four brain regions of the Goto-Kakizaki rat.** Raw reads correspond to the total number of reads obtained from the sequencer, and surviving reads the number of remaining reads after the trimming step. The percentage of surviving reads was calculated as the ratio of surviving reads to raw reads. Aligned reads are the number of reads aligned to the rat genome assembly Rno6.0. The percentage of aligned reads was calculated as the ratio of aligned reads to surviving reads. Alternative alignments indicate the number of duplicated read entries providing alternative coordinates, and the percentage was calculated as the ratio of alternative alignments to aligned reads. The mean coverage was determined as the ratio of the number of bp aligned to reference genome to the total number of bp in the reference. The exonic rate is the fraction of mapped reads within exons and the number of genes with at least 5 reads is given.

| **Sample** | **Raw reads** | **Surviving reads** | **%** | **Aligned reads** | **%** | **Alternative Alignments** | **%** | **Coverage** | **Exonic rate** | **Genes** |
| --- | --- | --- | --- | --- | --- | --- | --- | --- | --- | --- |
| Striatum_C6 | 101,208,846 | 100,166,480 | 99.0 | 98,266,468 | 98.1 | 9,369,985 | 9.5 | 66.13 | 0.64 | 17,578 |
| Striatum_C9 | 105,837,450 | 104,209,094 | 98.5 | 102,396,509 | 98.3 | 9,657,104 | 9.4 | 69.83 | 0.64 | 17,733 |
| Striatum_C7 | 101,303,036 | 100,013,238 | 98.7 | 98,229,606 | 98.2 | 9,216,101 | 9.4 | 63.64 | 0.64 | 17,614 |
| Striatum_C11 | 104,723,620 | 103,587,558 | 98.9 | 101,594,799 | 98.1 | 9,859,075 | 9.7 | 66.50 | 0.64 | 17,705 |
| Striatum_C13 | 71,849,470 | 70,879,158 | 98.6 | 69,609,271 | 98.2 | 6,867,013 | 9.9 | 42.04 | 0.64 | 17,191 |
| Striatum_C15 | 73,521,058 | 72,844,132 | 99.1 | 71,398,235 | 98.0 | 6,867,942 | 9.6 | 48.54 | 0.64 | 17,303 |
| Striatum_C12 | 98,826,102 | 97,770,476 | 98.9 | 95,976,626 | 98.2 | 9,636,424 | 10.0 | 60.79 | 0.63 | 17,657 |
| Striatum_C16 | 92,489,962 | 91,446,916 | 98.9 | 89,684,388 | 98.1 | 8,355,281 | 9.3 | 59.60 | 0.63 | 17,397 |
| Brainstem_F6 | 101,503,936 | 100,146,716 | 98.7 | 98,026,091 | 97.9 | 9,513,320 | 9.7 | 70.16 | 0.69 | 18,019 |
| Brainstem_F9 | 96,524,210 | 95,402,326 | 98.8 | 93,595,304 | 98.1 | 9,112,118 | 9.7 | 69.27 | 0.69 | 17,908 |
| Brainstem_F7 | 81,335,338 | 80,624,752 | 99.1 | 79,176,823 | 98.2 | 7,801,699 | 9.9 | 59.59 | 0.71 | 17,684 |
| Brainstem_F11 | 75,576,108 | 73,645,738 | 97.4 | 72,268,647 | 98.1 | 7,164,308 | 9.9 | 41.62 | 0.69 | 17,613 |
| Brainstem_F16 | 85,829,172 | 84,069,806 | 98.0 | 82,512,134 | 98.1 | 8,125,215 | 9.8 | 58.99 | 0.72 | 17,656 |
| Brainstem_F14 | 100,358,222 | 98,843,090 | 98.5 | 96,686,726 | 97.8 | 9,638,618 | 10.0 | 68.35 | 0.71 | 18,025 |
| Brainstem_F15 | 101,615,400 | 100,288,124 | 98.7 | 98,156,673 | 97.9 | 9,804,762 | 10.0 | 65.49 | 0.70 | 18,024 |
| Brainstem_F12 | 94,344,838 | 85,541,420 | 90.7 | 83,611,689 | 97.7 | 8,178,100 | 9.8 | 53.16 | 0.69 | 17,867 |
| Hypothalamus_A11 | 59,247,964 | 55,033,290 | 92.9 | 53,843,856 | 97.8 | 5,369,489 | 10.0 | 35.76 | 0.65 | 17,570 |
| Hypothalamus_A6 | 116,217,552 | 114,657,238 | 98.7 | 112,171,274 | 97.8 | 11,550,418 | 10.3 | 75.62 | 0.67 | 18,367 |
| Hypothalamus_A9 | 101,499,222 | 99,952,008 | 98.5 | 97,876,801 | 97.9 | 9,800,908 | 10.0 | 69.32 | 0.66 | 18,144 |
| Hypothalamus_A7 | 95,456,786 | 94,232,800 | 98.7 | 92,188,970 | 97.8 | 9,185,583 | 10.0 | 67.22 | 0.65 | 18,349 |
| Hypothalamus_A16 | 97,330,602 | 94,236,170 | 96.8 | 92,310,863 | 98.0 | 9,567,225 | 10.4 | 57.50 | 0.64 | 18,012 |
| Hypothalamus_A14 | 94,269,572 | 92,646,034 | 98.3 | 91,080,324 | 98.3 | 8,633,972 | 9.5 | 61.24 | 0.65 | 17,911 |
| Hypothalamus_A15 | 75,886,398 | 74,906,316 | 98.7 | 73,284,685 | 97.8 | 7,726,355 | 10.5 | 47.86 | 0.64 | 17,863 |
| Hypothalamus_A12 | 79,822,070 | 67,528,614 | 84.6 | 65,648,951 | 97.2 | 6,282,889 | 9.6 | 34.19 | 0.66 | 17,609 |
| Hippocampus_B11 | 53,328,018 | 52,544,866 | 98.5 | 51,463,669 | 97.9 | 4,720,713 | 9.2 | 38.32 | 0.65 | 17,015 |
| Hippocampus_B6 | 125,504,008 | 124,009,134 | 98.8 | 120,627,405 | 97.3 | 11,683,364 | 9.7 | 84.84 | 0.66 | 18,307 |
| Hippocampus_B9 | 79,796,930 | 78,778,678 | 98.7 | 77,272,774 | 98.1 | 7,158,100 | 9.3 | 59.18 | 0.66 | 17,621 |
| Hippocampus_B7 | 82,792,264 | 81,795,484 | 98.8 | 79,877,362 | 97.7 | 7,440,828 | 9.3 | 58.99 | 0.66 | 17,794 |
| Hippocampus_B15 | 74,313,806 | 73,521,510 | 98.9 | 72,083,520 | 98.0 | 6,643,381 | 9.2 | 53.93 | 0.66 | 17,373 |
| Hippocampus_B14 | 87,198,048 | 86,117,712 | 98.8 | 84,313,907 | 97.9 | 7,920,497 | 9.4 | 59.12 | 0.65 | 17,707 |
| Hippocampus_B16 | 90,231,800 | 89,229,428 | 98.9 | 87,136,215 | 97.7 | 8,240,592 | 9.5 | 64.28 | 0.65 | 17,733 |
| Hippocampus_B12 | 78,443,444 | 77,472,954 | 98.8 | 75,851,421 | 97.9 | 6,955,408 | 9.2 | 54.92 | 0.65 | 17,678 |
